# Surgical Pharmacy for Optimizing Medication Therapy Management Services within Enhanced Recovery after Surgery (ERAS^®^) Programs

**DOI:** 10.3390/jcm12020631

**Published:** 2023-01-12

**Authors:** Jingwen Xie, Xiaoyan Huang, Min Gao, Li Wei, Ruolun Wang, Jisheng Chen, Yingtong Zeng, Bo Ji, Tao Liu, Jinghao Wang, Hongwei Wu, Yong Wang, Li Qin, Yiting Wang, Zhuoling Zheng, Jing Xue, Junyan Wu, Xiao Chen, Zhihua Zheng, Xiaoyan Li

**Affiliations:** 1 Department of Pharmacy, The Sixth Affiliated Hospital, Sun Yat-sen University, Guangzhou 510655, China; 2 Department of Pharmacy, The First Affiliated Hospital of Guangzhou Medical University, Guangzhou 510120, China; 3 Department of Pharmacy, The Second Affiliated Hospital of Guangzhou Medical University, Guangzhou 510120, China; 4 Department of Pharmacy, The First Affiliated Hospital/School of Clinical Medicine of Guangdong Pharmaceutical University, Guangzhou 510080, China; 5 Department of Pharmacy, Guangdong Provincial People Hospital, Guangdong Academy of Medical Science, Guangzhou 510080, China; 6 Department of Clinical Pharmacy, General Hospital of Southern Theatre Command of People’s Liberation Army of China (PLA), Guangzhou 510010, China; 7 Department of Pharmacy, Sun Yat-sen University Cancer Center, Guangzhou 510060, China; 8 Department of Pharmacy, The First Affiliated Hospital of Jinan University, Guangzhou 510630, China; 9 Guangdong Province Pharmaceutical Association, Guangzhou 510080, China; 10 Department of Pharmacy, The Sun Yat-sen Memorial Hospital of Sun Yat-sen University, Guangzhou 510120, China; 11 Department of Pharmacy, The First Affiliated Hospital of Sun Yat-sen University, Guangzhou 510655, China

**Keywords:** surgical pharmacy, ERAS, pharmacist, perioperative medication therapy, work path

## Abstract

Drug-related problems (DRPs) are common among surgical patients, especially older patients with polypharmacy and underlying diseases. DRPs can potentially lead to morbidity, mortality, and increased treatment costs. The enhanced recovery after surgery (ERAS) system has shown great advantages in managing surgical patients. Medication therapy management for surgical patients (established as “surgical pharmacy” by Guangdong Province Pharmaceutical Association (GDPA)) is an important part of the ERAS system. Improper medication therapy management can lead to serious consequences and even death. In order to reduce DRPs further, and promote the rapid recovery of surgical patients, the need for pharmacists in the ERAS program is even more pressing. However, the medication therapy management services of surgical pharmacy and how surgical pharmacists should participate in ERAS programs are still unclear worldwide. Therefore, this article reviews the main perioperative medical management strategies and precautions from several aspects, including antimicrobial agents, antithrombotic agents, pain medication, nutritional therapy, blood glucose monitoring, blood pressure treatment, fluid management, treatment of nausea and vomiting, and management of postoperative delirium. Additionally, the way surgical pharmacists participate in perioperative medication management, and the relevant medication pathways are explored for optimizing medication therapy management services within the ERAS programs. This study will greatly assist surgical pharmacists’ work, contributing to surgeons accepting that pharmacists have an important role in the multidisciplinary team, benefitting medical workers in treating, counseling, and advocating for their patients, and further improving the effectiveness, safety and economy of medication therapy for patients and promoting patient recovery.

## 1. Introduction

Despite continuous advances in surgery, anesthesia, and perioperative care, undesirable complications during and after major surgery, such as pain, thrombogenesis, nausea, and gastrointestinal paralysis, continue to present major challenges. The enhanced recovery after surgery (ERAS) system has shown great advantages in managing surgical patients. This system refers to a series of optimized clinical pathways with evidence-based medicine (EBM) adopted in perioperative care to overcome the deleterious effect of perioperative stress, accelerate postoperative rehabilitation, reduce postoperative complications, shorten hospital stays, and reduce medical costs [1,2]. The concept of ERAS has spread to different surgical specialties and is widely used in patients receiving surgical operations. A multidisciplinary team (MDT), including surgery, anesthesia, pharmacy, nursing, rehabilitation, nutrition, and psychology, with team members made up of doctors, pharmacists, nurses, rehabilitation therapists, and dietitians, is required in the ERAS program, especially in cases of major surgery [2,3,4,5].

Medication therapy management is essential for surgery. Perioperative pain, nausea and vomiting, anticoagulation, anti-infection, blood pressure management, blood glucose management, nutrition management, fluid management and other aspects are all considered in medication therapy, as well as problems related to medication therapy. However, drug-related problems (DRPs) are common among hospitalized patients, potentially leading to morbidity, mortality, and increased treatment costs [6,7,8,9,10]. Patients attending surgical wards are especially at risk due to the need for pain medication, antibiotics, and frequent adjustments of antithrombotic regimens [8]. Mohammed et al., reported that up to 69.5% of patients had at least one DRP during their hospital stay among elective surgical patients [8]. In addition, polypharmacy is increasingly prevalent in older patients [11,12]. It was reported that polypharmacy occurred in 54.8% of older patients (≥65 years old) with elective noncardiac surgery [12]. Pharmaceutical interventions can significantly decrease DRPs and have an average cost savings of USD 1511 per case by identifying and resolving DRPs [13,14]. Therefore, in order to reduce DRPs, morbidity, and patient costs and promote the rapid recovery of surgical patients, there is a great need for the medication therapy management services of surgical pharmacy in the ERAS programs.

To date, surgical pharmacy has played an increasingly important role in the management of perioperative medication therapy. In 2015, the Guangdong Province Pharmaceutical Association (GDPA) first proposed the concept of a “surgical pharmacist” in China. In 2018, the GDPA officially applied to create the position of surgical pharmacist [15,16]. Then, in 2019, through our unremitting effort, the consensus of medical experts on the management of perioperative medication therapy in ERAS in China was published [17]. In 2021, the GDPA established a new discipline termed “surgical pharmacy”, which is the knowledge system of surgical pharmacists [18,19].

However, to date, medication therapy management services of surgical pharmacy and the way surgical pharmacists take part in ERAS programs are still unclear worldwide. Therefore, this paper reviews the main medical treatments and precautions for drug use in the perioperative period and explores how surgical pharmacists participate in perioperative medication management and develop relevant medication pathways for optimizing medication therapy management services within the ERAS programs.

## 2. Perioperative Medication Therapy

**Figure 1:**
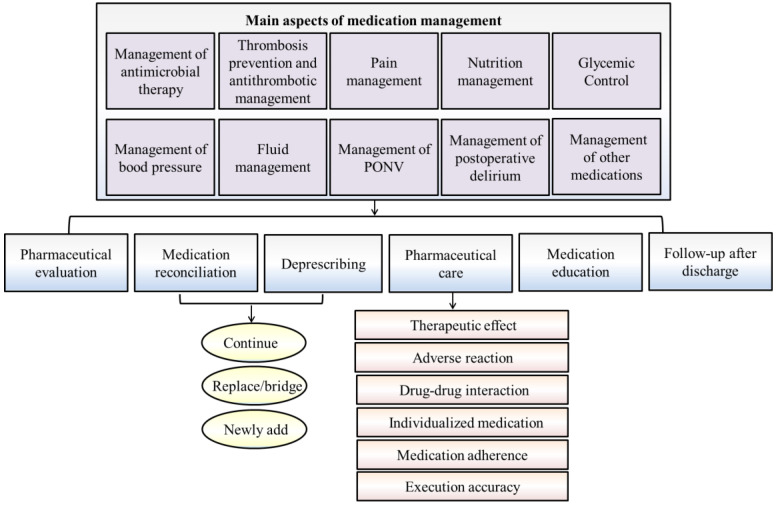
The main aspects of perioperative medication management and surgical pharmacists’ intervention within ERAS programs [17]. PONV: postoperative nausea and vomiting.

Perioperative medication therapy mainly includes antimicrobial agents, antithrombotic agents, pain medication, nutritional therapy, blood glucose monitoring, blood pressure treatment, fluid management, treatment of nausea and vomiting, and management of postoperative delirium. The aspects of surgical pharmacists’ involvement are summarized as follows (Figure 1), and the key points of medication monitoring are shown in Table 1.

### 2.1. Management of Antimicrobial Therapy

Surgical site infection (SSI) is a common postoperative complication and is the third most common nosocomial infection [75]. It was reported that SSI accounts for approximately 15–25% of all nosocomial infections and approximately 37% of the infections that occur in surgical patients [20,21,22]. The use of perioperative antibiotics is thought to be an important means to decrease wound infection. However, the irrational use of antibiotics can not only prolong the recovery time but also lead to the serious effects of antibiotic resistance [23].

According to the guiding principles for the clinical use of antibiotics (version 2015) [76], the use of prophylactic antibiotics should follow the principles of preventive medication and should be based on the type of surgical incision, the degree of surgical trauma, the type of possible contaminating bacteria, the duration of the operation, the chance of infection, the severity of the consequences, the levels of evidence for antimicrobial prophylaxis, the influence of drug resistance, economic evaluation and other factors. The need for the prophylactic medication of antibacterial drugs, appropriate antibacterial drugs and appropriate dosing regimens should be considered comprehensively.

### 2.2. Thrombosis Prophylaxis and Antithrombotic Management

Surgery is a well-recognized risk factor for thromboembolic disease. Since surgical patients are significantly more likely to develop venous thromboembolism (VTE) than ambulatory patients and experience higher rates of VTE recurrence and bleeding complications during VTE treatment, a trade-off must be considered in perioperative anticoagulant management. Existing evidence suggests that a targeted prophylaxis/treatment strategy based on patient-level variations would optimize the patient’s risk/benefit relationship and improve perioperative patient management [24]. However, the perioperative management of antithrombotic therapy, including anticoagulant and antiplatelet agents, often presents a dilemma for clinical practice. Although there is a relative paucity of well-designed clinical trials to inform the best perioperative practices in this area, most patients undergoing surgery are likely to benefit from pharmacologic prophylaxis [25]. The ninth edition of the ACCP guidelines on VTE risk assessment and prevention specifically recommends the Caprini score to quantify VTE risk and make prophylaxis recommendations for perioperative patients. The recommendations for perioperative management of thrombosis prophylaxis and antithrombotic therapy are shown in Table 1 [26,27,28,29,30].

### 2.3. Pain Management

Postoperative pain is acute pain that occurs immediately after surgery. Both undertreatment and overtreatment of acute postoperative pain can lead to severe consequences. Good pain management can reduce postoperative stress, accelerate the recovery of intestinal function, promote early recovery of patients, and improve the quality of life of patients after surgery [77]. Medication is essential in the treatment of pain. Postoperative analgesia needs to consider the following factors comprehensively: age, anxiety level, surgical method and process, individual body condition, and response to drugs or treatment [31,32,33,34,35]. During the perioperative period, the pain severity of patients and the efficacy of analgesic drugs should be dynamically assessed, and adverse reactions should be monitored. Analgesic drugs should be evaluated for adequacy and excess, and the medication regimen should be modified in time.

### 2.4. Nutrition Management

In surgical patients, especially in elderly individuals, malignant tumors, gastrointestinal diseases, and nervous system diseases are all commonly associated with a high malnutrition risk [36,78]. Nutritional status is an independent and effective indicator for predicting the incidence and mortality of perioperative complications [79,80,81]. In addition, the malnutrition risk in hospitalized patients was > 40% and higher after discharge [82]. Therefore, during the perioperative period, nutritional risk screening and assessment should be performed, and patients with nutritional risk should be given timely consideration and intervention.

### 2.5. Glycemic Control

Surgical patients frequently experience hyperglycemia, and undiagnosed insulin resistance is identified on the day of surgery (DOS) [83,84]. Surgical patients with diabetes are increasingly common and are more likely to present with glycemic control challenges [85]. Studies have shown that perioperative dysglycemia is associated with adverse postoperative clinical outcomes, including an increased incidence of postoperative infection, delayed wound healing, poor postoperative recovery and prolonged hospital stay, as well as an increased risk of surgery and perioperative mortality [84,86,87,88]. There is a 30% increased risk of adverse events for each 20-mg/dL increase in the mean glucose level [89,90]. Good glycemic control in the perioperative period is of great significance in improving the prognosis of patients.

### 2.6. Management of Blood Pressure

Perioperative blood pressure fluctuations directly affect the prognosis of patients. Good blood pressure control is of great significance for preventing intraoperative complications and improving the prognosis of patients. Abnormal fluctuations in perioperative blood pressure include hypertension and hypotension. Perioperative hypertension is usually caused by increased activity or insufficient inhibition of the autonomic nervous system and is related to patients’ emotions, such as tension and anxiety, primary hypertension, secondary hypertension, volume overload, anesthesia and other influencing factors [51]. Perioperative hypotension is relative to the patient’s basic blood pressure. It is related to the patient’s underlying diseases, the use of anesthesia or anesthetic drugs, neuroreflex hypotension, postural hypotension, supine hypotension syndrome, surgery and other factors, which can cause hypoperfusion of tissues and organs, and increase the risk of postoperative delirium, stroke, myocardial ischemia, myocardial infarction, acute kidney injury and postoperative mortality [52].

### 2.7. Fluid Management

The normal metabolism of water, electrolytes and acid-base balance in the human body are important factors in maintaining the stability of the body’s internal environment, which is an indispensable condition for the normal life activities of the body. Insufficient infusion can cause hypoperfusion, microcirculation disorders, and organ insufficiency in patients with heart, kidney, brain and other vital organs. Excessive infusion can cause postoperative intra-abdominal hypertension and interstitial edema, affect the healing of anastomosis and the recovery of gastrointestinal function, and increase the probability of systemic infection, both of which can lead to increased postoperative morbidity and mortality [55,56].

The goal of perioperative fluid therapy in the ERAS protocol is to maintain the homeostasis of body fluids and avoid postoperative complications and gastrointestinal dysfunction due to fluid overload or organ insufficiency. In the perioperative period, a goal-directed circulatory management strategy is advocated, especially for complex surgery and critically ill patients [57].

### 2.8. Management of Postoperative Nausea and Vomiting

Postoperative nausea and vomiting (PONV) were found in approximately 30% of general surgical patients and as high as 80% of high-risk patients, which can lead to electrolyte disorders, wound dehiscence, esophageal rupture and delayed discharge time [91]. It was reported that risk factors for PONV included age (<50 years old), female sex, nonsmoker, history of motion sickness or PONV, opioid analgesia and surgery type [59]. For the selection and use of PONV drugs, the risk of PONV should first be assessed in patients. It was recommended that multimodal prophylaxis be used in patients with one or more risk factors [60]. In ERAS pathways, multimodal prophylactic antiemetics are recommended [60].

### 2.9. Management of Postoperative Delirium

Delirium is a common and harrowing complication during the postoperative period, especially in older patients. Postoperative delirium occurs in 17–61% of major surgeries, which may cause cognitive decline, prolonged length of stay (LOS), decreased functional independence, increased risk of dementia, caregiver burden, health care costs, morbidity and mortality [92,93]. Older age, dementia (often not recognized clinically), frailty, functional disabilities, the severity of concurrent illness, type of operation, ICU admission after surgery, a high burden of coexisting conditions and postoperative pain are common predisposing factors [64,65]. Male sex, poor vision and hearing, depressive symptoms, mild cognitive impairment, laboratory abnormalities, and alcohol abuse have also been associated with increased risk [66]. Delirium can be prevented, and 30–40% of cases are assumed to be preventable before its onset [94]. Pharmacological prevention and treatment are important aspects.

## 3. How Surgical Pharmacy Is Engaged in Perioperative Medication Management

As shown above, medication therapy involves all aspects of the preoperative and postoperative periods and includes special management of certain drugs, such as anesthetics, psychotics, radiopharmaceuticals, off-label medication, and proton pump inhibitors (shown in Figure 1) [17]. The key points of medication monitoring are depicted in Table 1. Surgical pharmacists are indispensable as ERAS team members; they can focus on patients to formulate clinical drug treatment strategies, prescribe rational drug use based on medication treatment management, pharmaceutical evaluation and monitoring of patients with underlying diseases, manage patients throughout the perioperative period, and coordinate multidisciplinary comprehensive diagnosis to promote recovery from surgery. There is evolving literature that proves the collaborative contributions of pharmacists in selecting pharmacotherapy or alternative drugs, minimizing misuse or overuse of medications, contributing to improved outcomes, reducing complications and decreasing costs and thus shortening LOS [95,96,97,98,99,100,101,102]. The detailed entry points for surgical pharmacists to participate in perioperative ERAS medication therapy management are as follows [17]:

### 3.1. Make an Estimate of Medication Deprescribing and Therapy-Related Problems before Surgery

The pharmacist performs a pharmaceutical assessment of the patient before hospitalization or admission and obtains a complete medication and allergy history, including medication purpose, drug name (generic name, trade name), specifications, usage and dosage, and medication course [103]. The medication list is collected and analyzed, and the patient’s previous medication is compared with the prescribed pre-surgery medication. The pharmacist considers the need to discontinue the use of drugs, drug interactions, and repeated drug use and analyzes the pharmacokinetics, followed by reforming and simplifying the prescription and making a list of medication interventions and recommendations. In addition, surgical pharmacists should clarify the content of perioperative drug monitoring and make a detailed monitoring plan.

### 3.2. Implement ERAS Standardized Medication Treatment Path during the Perioperative Period

A list of the common surgery-related drugs is formulated. Considering different diseases, surgical types, special populations and other factors, the process and focus of medication therapy management should be adjusted individually, and the whole-course medication therapy plan and working pathway of ERAS suitable for the hospital should be developed jointly with doctors [104,105]. In addition, surgical pharmacists can provide drug consultation and training to ERAS teams from the perspective of drug treatment-related issues, such as drug safety, drug interactions, adverse drug reactions, and drug use characteristics in specific populations. At the same time, surgical pharmacists will evaluate the efficacy of medication therapy to jointly improve the team’s medication therapy level in the implementation of ERAS.

### 3.3. Hospital Medication Education and Follow-Up

Surgical pharmacists can conduct postoperative medication education for patients according to the specific disease type, postoperative category and key use of drugs, the efficacy indicators of medication therapy, adverse reactions and medication compliance. Unified follow-up plans and forms should be developed, which should be diversified and individualized based on specific diseases and medications.

## 4. Workflow and Work Path of Medication Treatment Management

**Figure 2:**
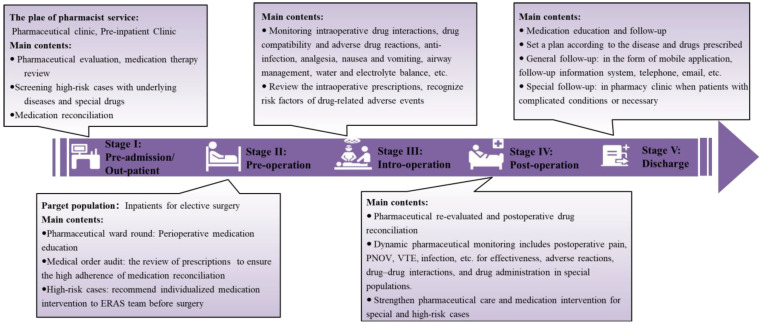
Workflow chart for surgical pharmacists in perioperative medication treatment management within ERAS programs. PONV: postoperative nausea and vomiting; VTE, venous thromboembolism.

The whole-process management of perioperative medication therapy in ERAS requires pharmacists to participate in the pharmaceutical evaluation, medication reconciliation, deprescribing, pharmaceutical care, medication education and follow-up after discharge [106]. After a discussion with authoritative pharmaceutical experts in this field in China, the workflow and work path was finally determined. Therefore, the scope of pharmacist participation in ERAS medication treatment management can be divided into five stages [17]: stage I: Pre-admission/outpatient pharmaceutical care; stage II: Preoperative pharmaceutical evaluation and service; stage III: Intraoperative pharmaceutical care; stage IV: Postoperative pharmaceutical assessment and care; stage V: Medication education and follow-up for the discharge. The specific workflow chart for surgical pharmacists within the ERAS programs is shown in Figure 2.

### 4.1. Stage I: Pre-Admission/Out-Patient Pharmaceutical Care

Before admission, surgical pharmacists will take a medication therapy review and collect patients’ personal medication history in detail before surgery, according to the specific situation, to conduct drug reconciliation [107], drug discontinuation/bridging/replacement therapy, deprescribing, provide pharmaceutical recommendations for ERAS short-term drug protocols individualized for each patient and generate pre-evaluation records [108]. The pre-evaluation of recommendations for medication is communicated to the ERAS team, and the next step is determined through a comprehensive clinical assessment.

### 4.2. Stage II: Preoperative Pharmaceutical Assessment and Service

A pharmaceutical assessment is conducted for patients admitted for elective surgery after pre-admission evaluation, which is throughout the whole process of ERAS. The key points of preoperative pharmaceutical care include (1) Preoperative medication education for patients; (2) The review of prescriptions based on outpatient pharmacy pre-assessment records to ensure high adherence to medication reconciliation [109]; and (3) For high-risk cases in preoperative assessment, individualized drug intervention plans are recommended to the ERAS treatment team before surgery to optimize ERAS medication treatment [106,110].

### 4.3. Stage III: Intraoperative Pharmaceutical Care

The patients are typically in the operating room under anesthesia during surgery, and the key points of intraoperative pharmaceutical care include the following: (1) Appropriate indications, variety and timing of antibacterial drugs applied in the operating room; (2) Knowledge of intraoperative drug interactions, drug compatibility and adverse drug reactions, with emphasis on monitoring analgesic drugs, analgesic pump application, airway management and water and electrolyte balance; (3) Review of postoperative medication prescriptions and recognition of risk factors of underlying drug-related adverse events.

### 4.4. Stage IV: Postoperative Pharmacy Reassessment and Monitoring

Patients need to be involved in pharmaceutical care after surgery. Medication management includes assessment of postoperative pain, nausea and vomiting monitoring [110], nutrition status, venous thromboembolism, infection prevention and treatment, and the reasons for poor efficacy, safety, adherence [111], and execution accuracy of ERAS medication therapy. Postoperative medication reconciliation and dynamic pharmaceutical monitoring are instituted for drug treatment effects, adverse reactions, drug–drug interactions, and drug administration in special populations.

### 4.5. Stage V: Medication Education and Follow-up after Discharge

Medication education for postoperative patients can be provided according to the type of disease, operation, drug and follow-up time. A plan can be set according to the disease and drugs prescribed, and follow-up can be performed in the form of mobile application [112,113], pharmacy clinic [114], telephone, email and other forms of follow-up information systems [115,116].

## 5. Conclusions

Surgical patients are typically administered a wide variety of medications, and the surgery itself, underlying diseases, and preoperative or postoperative treatments affect the pharmacokinetics. The quality of medical care for surgical patients is not only related to the surgery itself but also closely related to medication. Postoperative complications, comorbidities and improper medication can lead to serious consequences and even death of the patients. Pharmacists have played an important role in recognizing DRPs and medication management. This article will be beneficial for guiding surgical pharmacists to carry out their work in an ERAS team. Surgical pharmacists can participate in perioperative medication therapy management, formulate patient-centered clinical medication therapy strategies, and provide medication therapy management dominated by rational drug use to improve the effectiveness, safety and economy of medication therapy for patients and promote medical safety. In addition, this review contributes to surgeons accepting pharmacists as having an important role in the multidisciplinary team, assisting medical workers in treating, counseling, and advocating for their patients.

However, the successful implementation of surgical pharmacy services within the ERAS programs requires the patients’ and surgical pharmacists’ active participation. In recent years, we have established the system of surgical pharmacy, compiled the teaching materials of surgical pharmacy, and carried out the training of surgical pharmacy nationwide to strengthen the mastery and application of professional knowledge of surgical pharmacy by pharmacists. During our work, a habit and protocol of record keeping (preferably electronic) need to be developed to allow a regular periodic audit of the outcomes and guide further learning and continuous improvement. In addition, future research with more detailed descriptions to evaluate the impact of each of the pharmacists’ interventions is necessary in order to guide pharmacists and medical workers. The problems found should be summarized in time and solved to form a virtuous cycle, and more details items of medication therapy management within ERAS programs should be refined continuously.

## Figures and Tables

**Table 1:** Representative drugs commonly used during the perioperative period and key points of medication monitoring.

Main Aspects	Key Points of Medication Monitoring
Management of antimicrobial therapy [20,21,22,23]	Firmly grasp the principle of preventive and treating medicine; Species selection of prophylactic and treatment medication; Factors to be considered in the formulation of medication regiments: the category of the incision, the degree of surgical trauma, possible pollution type, the duration of surgery, the incidence of infection, the severity of the infection, antibacterial drug prevention and treating effect, the effective concentration of drugs at the surgical site, the bacterial drug resistance, safety, economic factors. Try to choose a single antibacterial drug for prophylaxis; Timing of prophylactic medication: intravenous infusion should be administered within 0.5–1 h before skin and mucous membrane incision or at the beginning of anesthesia, and the operation should be started after the infusion; Prophylactic medication course: the duration of effective antimicrobial coverage should include the entire surgical procedure.
Thrombosis prevention and antithrombotic management [24,25,26,27,28,29,30]	Dynamically assess the patient’s risk of bleeding and VTE; For patients at high risk of VTE, drug prophylaxis is the first choice if there are no contraindications; Determine the timing of drug withdrawal before surgery according to the half-life of the drug; Determine the timing of postoperative drug resumption according to the onset time of the drug; Patients receiving long-acting antithrombotic drugs can receive appropriate antithrombotic drugs as an alternative therapy before surgery; The benefits of perioperative bridging vary with different types of antithrombotic agents.
Pain management [31,32,33,34,35]	Multimodal analgesia is recommended; Surgical pharmacists should provide the following: (1) Pain assessment and medication safety education for patients with perioperative pain; (2) Opioid conversion, evaluation of evidence-based medical strategy and other pharmacy services; (3) Pain health education and analgesic medication advice; (4) Quality control of pain assessment. 3. There are many gastrointestinal side effects associated with nonselective NSAID preventive analgesia, especially for patients prescribed anticoagulants, aspirin, glucocorticoids or who have a history of gastrointestinal ulcer H2 receptor antagonists; PPI or selective COX2 inhibitors can be used.
Nutrition management [36,37,38,39,40,41,42]	Nutritional risk screening and assessment should be performed, and patients with nutritional risk should be given timely interventions; Understand the metabolic changes in the body under various conditions, choose reasonable nutritional support approaches, provide suitable nutritional substrates, and avoid or reduce the occurrence of complications as much as possible; Oral nutritional supplement (ONS) or enteral nutrition (EN) is the first choice for ERAS perioperative nutritional support; When EN cannot be implemented or provide sufficient capacity and protein, parenteral nutrition (PN) should be supplemented or selected.
Glycemic control [43,44,45,46,47,48,49,50]	Provide dynamic evaluation of patients with perioperative dysglycemia based on the type of surgery, patient conditions, and medication use during hospitalization; Conduct pharmaceutical care for patients with abnormal blood glucose, pay attention to the influence of drugs on blood glucose or the interaction with hypoglycemic drugs and perform drug restructuring. Develop individual blood glucose control goals, treatment plans and blood glucose monitoring plans; Early morning surgery is recommended for people with diabetes to minimize the impact of fasting on blood glucose control; Insulin is the preferred treatment for perioperative glycemic control; Patients treated with oral hypoglycemic agents or noninsulin injectable agents (GLP-1 analogs) are advised to discontinue the original regimen on the morning of surgery; Most hypoglycemic drugs can continue to be used following the original treatment plan after patients return to a normal diet after surgery.
Management of blood pressure [51,52,53,54]	Closely monitor hemodynamics during the perioperative period to avoid large fluctuations in blood pressure and the occurrence of hypotension; For blood pressure management, individualized blood pressure control goals and treatment plans should be formulated according to the specific situation of patients, the degree of disease, the type of surgery; Preoperative discontinuation of calcium channel blockers is not recommended, and initiation of calcium channel blockers may be considered for patients who cannot tolerate β –blockers; ACEI should be discontinued or reduced before surgery, while ARB should be discontinued on the day of surgery or before surgery until the fluid volume is restored; Preoperative withdrawal of diuretics is recommended; For patients with a history of hypertension, the risk of hypotension during the perioperative period is much higher than hypertension, and sympathomimetic drugs such as norepinephrine and dopamine can be used to increase blood pressure; Particular attention should be given to the effects of perioperative analgesics and anesthetics on blood pressure.
Fluid management [55,56,57,58]	1. According to different therapeutic purposes, disease states and stages, a reasonable fluid treatment plan is formulated and implemented individually; 2. Maintain the homeostasis of body fluids and avoid postoperative complications and gastrointestinal dysfunction due to fluid overload or organ insufficiency; 3. For patients who have insufficient blood volume and need a large amount of fluid replacement, it is recommended to supplement the crystalloid solution and infuse the colloidal solution appropriately to control the infusion volume and reduce tissue edema; 4. For patients who do not have hypovolemia (only extracellular fluid or functional extracellular fluid), it is recommended to supplement the physiological requirement with a crystalloid solution; 5. For critically ill patients who require a large amount of fluid resuscitation, especially when complicated with acute lung injury, it is recommended to choose albumin for goal-directed restrictive fluid therapy.
Management of postoperative nausea and vomiting (PONV) [59,60,61,62,63]	1. Identify the patient’s risk for PONV and take corresponding preventive measures according to the patient’s risk grade; 2. Use multimodal prophylaxis in patients with one or more risk factors; 3. Administer PONV prophylaxis Using two agents in adults at 1–2 risks for PONV; 4. Administer PONV prophylaxis Using four agents in adults at > 2 risks for PONV; 5. For patients who do not receive PONV prophylaxis, low-dose 5-HT3 receptor antagonist therapy remains the first line for dealing with the occurrence of PONV; 6. If PONV prophylaxis fails, drugs with different mechanisms of action may be used for prophylaxis.
Management of postoperative delirium (POD) [64,65,66,67,68,69,70,71,72,73,74]	Identify risk factors for POD; Central anticholinergic drugs, benzodiazepines, and pethidine should be avoided; Current first-choice pharmacologic agents for delirium treatment are antipsychotic medications, including haloperidol, olanzapine, and quetiapine; Dexmedetomidine is also being used as a primary therapy for delirium.

## Data Availability

Not applicable.

## References

[1] Klek S., Salowka J., Choruz R., Cegielny T., Welanyk J., Wilczek M., Szczepanek K., Pisarska-Adamczyk M., Pedziwiatr M. (2021). Enhanced Recovery after Surgery (ERAS) Protocol Is a Safe and Effective Approach in Patients with Gastrointestinal Fistulas Undergoing Reconstruction: Results from a Prospective Study. Nutrients.

[2] Ljungqvist O., Scott M., Fearon K.C. (2017). Enhanced Recovery after Surgery: A Review. JAMA Surg..

[3] Wang D., Liu Z., Zhou J., Yang J., Chen X., Chang C., Liu C., Li K., Hu J. (2022). Barriers to implementation of enhanced recovery after surgery (ERAS) by a multidisciplinary team in China: A multicentre qualitative study. BMJ Open.

[4] Francis N.K., Walker T., Carter F., Hubner M., Balfour A., Jakobsen D.H., Burch J., Wasylak T., Demartines N., Lobo D.N. (2018). Consensus on Training and Implementation of Enhanced Recovery after Surgery: A Delphi Study. World J. Surg..

[5] Roulin D., Najjar P., Demartines N. (2017). Enhanced Recovery after Surgery Implementation: From Planning to Success. J. Laparoendosc. Adv. Surg. Tech..

[6] Hoonhout L.H., de Bruijne M.C., Wagner C., Asscheman H., van der Wal G., van Tulder M.W. (2010). Nature, occurrence and consequences of medication-related adverse events during hospitalization: A retrospective chart review in the Netherlands. Drug Saf..

[7] Krahenbuhl-Melcher A., Schlienger R., Lampert M., Haschke M., Drewe J., Krahenbuhl S. (2007). Drug-related problems in hospitals: A review of the recent literature. Drug Saf..

[8] Mohammed M., Bayissa B., Getachew M., Adem F. (2022). Drug-related problems and determinants among elective surgical patients: A prospective observational study. SAGE Open Med..

[9] Davies E.C., Green C.F., Taylor S., Williamson P.R., Mottram D.R., Pirmohamed M. (2009). Adverse drug reactions in hospital in-patients: A prospective analysis of 3695 patient-episodes. PloS ONE.

[10] Miguel A., Azevedo L.F., Araujo M., Pereira A.C. (2012). Frequency of adverse drug reactions in hospitalized patients: A systematic review and meta-analysis. Pharmacoepidemiol. Drug Saf..

[11] Barlow A., Prusak E.S., Barlow B., Nightingale G. (2021). Interventions to reduce polypharmacy and optimize medication use in older adults with cancer. J. Geriatr. Oncol..

[12] McIsaac D.I., Wong C.A., Bryson G.L., van Walraven C. (2018). Association of Polypharmacy with Survival, Complications, and Healthcare Resource Use after Elective Noncardiac Surgery: A Population-based Cohort Study. Anesthesiology.

[13] Bos J.M., van den Bemt P.M., Kievit W., Pot J.L., Nagtegaal J.E., Wieringa A., van der Westerlaken M.M., van der Wilt G.J., de Smet P.A., Kramers C. (2017). A multifaceted intervention to reduce drug-related complications in surgical patients. Br. J. Clin. Pharmacol..

[14] Dagnew S.B., Binega Mekonnen G., Gebeye Zeleke E., Agegnew Wondm S., Yimer Tadesse T. (2022). Clinical Pharmacist Intervention on Drug-Related Problems among Elderly Patients Admitted to Medical Wards of Northwest Ethiopia Comprehensive Specialized Hospitals: A Multicenter Prospective, Observational Study. BioMed Res. Int..

[15] Zheng Z., Wu J., Zeng Y., Wang R., Wang J., Li X., Li J., Chen W., Wang Y. (2020). Promoting the establishment of the position of “surgical pharmacist”. Pharm. Today.

[16] Zheng Z., Wu J., Zeng Y., Wang R., Wang J., Wang Y. (2020). Creating the position of surgical pharmacist in China. Eur. J. Hosp. Pharm..

[17] Guangdong Province Pharmaceutical Association (2020). Consensus of medical experts on the management of perioperative medication therapy in ERAS. Pharm. Today.

[18] Wu J., Zhang M., Wang R., Wei L., Li X., Zeng Y., Chen J., Ji B., Wu H., Wang J. (2021). Surgical pharmacy: Knowledge construction for surgical pharmacists. Pharm. Today.

[19] Zheng Z., Wu J., Wei L., Li X., Ji B., Wu H. (2023). Surgical pharmacy: The knowledge system of surgical pharmacists. Eur. J. Hosp. Pharm..

[20] Bratzler D.W., Houck P.M., Surgical Infection Prevention Guideline Writers W. (2005). Antimicrobial prophylaxis for surgery: An advisory statement from the National Surgical Infection Prevention Project. Am. J. Surg..

[21] Kaiser A.B. (1991). Surgical-wound infection. N. Engl. J. Med..

[22] Young P.Y., Khadaroo R.G. (2014). Surgical site infections. Surg. Clin. N. Am..

[23] Allen M.S. (2005). Perioperative antibiotics: When, why?. Thorac. Surg. Clin..

[24] Farge D., Frere C., Connors J.M., Khorana A.A., Kakkar A., Ay C., Munoz A., Brenner B., Prata P.H., Brilhante D. (2022). 2022 international clinical practice guidelines for the treatment and prophylaxis of venous thromboembolism in patients with cancer, including patients with COVID-19. Lancet. Oncol..

[25] Liu M., Wang G., Li Y., Wang H., Liu H., Guo N., Han C., Peng Y., Yang M., Liu Y. (2020). Efficacy and safety of thromboprophylaxis in cancer patients: A systematic review and meta-analysis. Ther. Adv. Med. Oncol..

[26] Akl E.A., Vasireddi S.R., Gunukula S., Barba M., Sperati F., Terrenato I., Muti P., Schunemann H. (2011). Anticoagulation for the initial treatment of venous thromboembolism in patients with cancer. Cochrane Database Syst. Rev..

[27] Hornor M.A., Duane T.M., Ehlers A.P., Jensen E.H., Brown P.S., Pohl D., da Costa P.M., Ko C.Y., Laronga C. (2018). American College of Surgeons’ Guidelines for the Perioperative Management of Antithrombotic Medication. J. Am. Coll. Surg..

[28] Kristensen S.D., Knuuti J., Saraste A., Anker S., Botker H.E., De Hert S., Ford I., Gonzalez Juanatey J.R., Gorenek B., Heyndrickx G.R. (2014). 2014 ESC/ESA Guidelines on non-cardiac surgery: Cardiovascular assessment and management: The Joint Task Force on non-cardiac surgery: Cardiovascular assessment and management of the European Society of Cardiology (ESC) and the European Society of Anaesthesiology (ESA). Eur. J. Anaesthesiol..

[29] Narouze S., Benzon H.T., Provenzano D., Buvanendran A., De Andres J., Deer T., Rauck R., Huntoon M.A. (2018). Interventional Spine and Pain Procedures in Patients on Antiplatelet and Anticoagulant Medications (Second Edition): Guidelines from the American Society of Regional Anesthesia and Pain Medicine, the European Society of Regional Anaesthesia and Pain Therapy, the American Academy of Pain Medicine, the International Neuromodulation Society, the North American Neuromodulation Society, and the World Institute of Pain. Reg. Anesth. Pain Med..

[30] Diener H.C., Cunha L., Forbes C., Sivenius J., Smets P., Lowenthal A. (1996). European Stroke Prevention Study. 2. Dipyridamole and acetylsalicylic acid in the secondary prevention of stroke. J. Neurol. Sci..

[31] Mitra S., Carlyle D., Kodumudi G., Kodumudi V., Vadivelu N. (2018). New Advances in Acute Postoperative Pain Management. Curr. Pain Headache Rep..

[32] Joshi G.P., Kehlet H. (2019). Postoperative pain management in the era of ERAS: An overview. Best Pract. Res. Clin. Anaesthesiol..

[33] Tan M., Law L.S., Gan T.J. (2015). Optimizing pain management to facilitate Enhanced Recovery after Surgery pathways. Can. J. Anaesth..

[34] Amaechi O., Huffman M.M., Featherstone K. (2021). Pharmacologic Therapy for Acute Pain. Am. Fam. Physician.

[35] Blondell R.D., Azadfard M., Wisniewski A.M. (2013). Pharmacologic therapy for acute pain. Am. Fam. Physician.

[36] Weimann A., Braga M., Carli F., Higashiguchi T., Hubner M., Klek S., Laviano A., Ljungqvist O., Lobo D.N., Martindale R.G. (2021). ESPEN practical guideline: Clinical nutrition in surgery. Clin. Nutr..

[37] Wischmeyer P.E., Carli F., Evans D.C., Guilbert S., Kozar R., Pryor A., Thiele R.H., Everett S., Grocott M., Gan T.J. (2018). American Society for Enhanced Recovery and Perioperative Quality Initiative Joint Consensus Statement on Nutrition Screening and Therapy within a Surgical Enhanced Recovery Pathway. Anesth. Analg..

[38] Wobith M., Weimann A. (2021). Oral Nutritional Supplements and Enteral Nutrition in Patients with Gastrointestinal Surgery. Nutrients.

[39] Weimann A., Braga M., Carli F., Higashiguchi T., Hubner M., Klek S., Laviano A., Ljungqvist O., Lobo D.N., Martindale R. (2017). ESPEN guideline: Clinical nutrition in surgery. Clin. Nutr..

[40] Elke G., van Zanten A.R., Lemieux M., McCall M., Jeejeebhoy K.N., Kott M., Jiang X., Day A.G., Heyland D.K. (2016). Enteral versus parenteral nutrition in critically ill patients: An updated systematic review and meta-analysis of randomized controlled trials. Crit. Care.

[41] Abunnaja S., Cuviello A., Sanchez J.A. (2013). Enteral and parenteral nutrition in the perioperative period: State of the art. Nutrients.

[42] Hellerman Itzhaki M., Singer P. (2020). Advances in Medical Nutrition Therapy: Parenteral Nutrition. Nutrients.

[43] Cheisson G., Jacqueminet S., Cosson E., Ichai C., Leguerrier A.M., Nicolescu-Catargi B., Ouattara A., Tauveron I., Valensi P., Benhamou D. (2018). Perioperative management of adult diabetic patients. Intraoperative period. Anaesth. Crit. Care Pain Med..

[44] American Diabetes A. (2018). 14. Diabetes Care in the Hospital: Standards of Medical Care in Diabetes-2018. Diabetes Care.

[45] Dhatariya K., Levy N., Kilvert A., Watson B., Cousins D., Flanagan D., Hilton L., Jairam C., Leyden K., Lipp A. (2012). NHS Diabetes guideline for the perioperative management of the adult patient with diabetes. Diabet. Med. J. Br. Diabet. Assoc..

[46] Palermo N.E., Garg R. (2019). Perioperative Management of Diabetes Mellitus: Novel Approaches. Curr. Diabetes Rep..

[47] Booth G., Cheng A.Y., Canadian Diabetes Association Clinical Practice Guidelines Expert Committee (2013). Canadian Diabetes Association 2013 clinical practice guidelines for the prevention and management of diabetes in Canada. Methods. Can. J. Diabetes.

[48] David J.S., Tavernier B., Amour J., Vivien B., Coriat P., Riou B. (2004). Myocardial effects of halothane and sevoflurane in diabetic rats. Anesthesiology.

[49] Kadoi Y. (2012). Blood glucose control in the perioperative period. Minerva Anestesiol..

[50] Deacon S.P., Karunanayake A., Barnett D. (1977). Acebutolol, atenolol, and propranolol and metabolic responses to acute hypoglycaemia in diabetics. Br. Med. J..

[51] Saugel B., Sessler D.I. (2021). Perioperative Blood Pressure Management. Anesthesiology.

[52] Saugel B., Kouz K., Hoppe P., Maheshwari K., Scheeren T.W.L. (2019). Predicting hypotension in perioperative and intensive care medicine. Best Pract. Res. Clin. Anaesthesiol..

[53] Sousa-Uva M., Milojevic M., Head S.J., Jeppsson A. (2018). The 2017 EACTS guidelines on perioperative medication in adult cardiac surgery and patient blood management. Eur. J. Cardio-Thorac. Surg..

[54] San Roman J.A., Spanish Society of Cardiology Working Group for the the 2014 ESC/ESA Guidelines on Non-Cardiac Surgery, Expert Reviewers for the 2014 ESC/ESA Guidelines on Non-Cardiac Surgery, Clinical Practice Guidelines Committee of the Spanish Society of Cardiology (2014). Comments on the 2014 ESC/ESA Guidelines on Noncardiac Surgery: Cardiovascular assessment and management. Rev. Esp. Cardiol..

[55] Brandstrup B., Tønnesen H., Beier-Holgersen R., Hjortsø E., Ørding H., Lindorff-Larsen K., Rasmussen M.S., Lanng C., Wallin L., Iversen L.H. (2003). Effects of intravenous fluid restriction on postoperative complications: Comparison of two perioperative fluid regimens: A randomized assessor-blinded multicenter trial. Ann. Surg..

[56] Asklid D., Segelman J., Gedda C., Hjern F., Pekkari K., Gustafsson U.O. (2017). The impact of perioperative fluid therapy on short-term outcomes and 5-year survival among patients undergoing colorectal cancer surgery—A prospective cohort study within an ERAS protocol. Eur. J. Surg. Oncol. J. Eur. Soc. Surg. Oncol. Br. Assoc. Surg. Oncol..

[57] Scheib S.A., Thomassee M., Kenner J.L. (2019). Enhanced Recovery after Surgery in Gynecology: A Review of the Literature. J. Minim. Invasive Gynecol..

[58] Navarro L.H., Bloomstone J.A., Auler J.O., Cannesson M., Rocca G.D., Gan T.J., Kinsky M., Magder S., Miller T.E., Mythen M. (2015). Perioperative fluid therapy: A statement from the international Fluid Optimization Group. Perioper. Med..

[59] Apfel C.C., Heidrich F.M., Jukar-Rao S., Jalota L., Hornuss C., Whelan R.P., Zhang K., Cakmakkaya O.S. (2012). Evidence-based analysis of risk factors for postoperative nausea and vomiting. Br. J. Anaesth..

[60] Gan T.J., Belani K.G., Bergese S., Chung F., Diemunsch P., Habib A.S., Jin Z., Kovac A.L., Meyer T.A., Urman R.D. (2020). Fourth Consensus Guidelines for the Management of Postoperative Nausea and Vomiting. Anesth. Analg..

[61] Choi Y.S., Sohn H.M., Do S.H., Min K.T., Woo J.H., Baik H.J. (2018). Comparison of ramosetron and ondansetron for the treatment of established postoperative nausea and vomiting after laparoscopic surgery: A prospective, randomized, double-blinded multicenter trial. Ther. Clin. Risk Manag..

[62] Deitrick C.L., Mick D.J., Lauffer V., Prostka E., Nowak D., Ingersoll G. (2015). A comparison of two differing doses of promethazine for the treatment of postoperative nausea and vomiting. J. Perianesthesia Nurs..

[63] Kazemi-Kjellberg F., Henzi I., Tramèr M.R. (2001). Treatment of established postoperative nausea and vomiting: A quantitative systematic review. BMC Anesthesiol..

[64] Janssen T.L., Alberts A.R., Hooft L., Mattace-Raso F., Mosk C.A., van der Laan L. (2019). Prevention of postoperative delirium in elderly patients planned for elective surgery: Systematic review and meta-analysis. Clin. Interv. Aging.

[65] Johnson J.R. (2018). Delirium in Hospitalized Older Adults. N. Engl. J. Med..

[66] Allen S.R., Frankel H.L. (2012). Postoperative complications: Delirium. Surg. Clin. N. Am..

[67] Hughes C.G., Boncyk C.S., Culley D.J., Fleisher L.A., Leung J.M., McDonagh D.L., Gan T.J., McEvoy M.D., Miller T.E., Perioperative Quality Initiative W. (2020). American Society for Enhanced Recovery and Perioperative Quality Initiative Joint Consensus Statement on Postoperative Delirium Prevention. Anesth. Analg..

[68] Santos E., Cardoso D., Neves H., Cunha M., Rodrigues M., Apostolo J. (2017). Effectiveness of haloperidol prophylaxis in critically ill patients with a high risk of delirium: A systematic review. JBI Database Syst. Rev. Implement. Rep..

[69] Liu Y., Ma L., Gao M., Guo W., Ma Y. (2016). Dexmedetomidine reduces postoperative delirium after joint replacement in elderly patients with mild cognitive impairment. Aging Clin. Exp. Res..

[70] Gamberini M., Bolliger D., Lurati Buse G.A., Burkhart C.S., Grapow M., Gagneux A., Filipovic M., Seeberger M.D., Pargger H., Siegemund M. (2009). Rivastigmine for the prevention of postoperative delirium in elderly patients undergoing elective cardiac surgery—A randomized controlled trial. Crit. Care Med..

[71] Liptzin B., Laki A., Garb J.L., Fingeroth R., Krushell R. (2005). Donepezil in the prevention and treatment of post-surgical delirium. Am. J. Geriatr. Psychiatry.

[72] Rengel K.F., Pandharipande P.P., Hughes C.G. (2018). Postoperative delirium. Presse Med..

[73] Carrasco G., Baeza N., Cabre L., Portillo E., Gimeno G., Manzanedo D., Calizaya M. (2016). Dexmedetomidine for the Treatment of Hyperactive Delirium Refractory to Haloperidol in Nonintubated ICU Patients: A Nonrandomized Controlled Trial. Crit. Care Med..

[74] Cavallaro P., Bordeianou L. (2019). Implementation of an ERAS Pathway in Colorectal Surgery. Clin. Colon Rectal Surg..

[75] Mangram A.J., Horan T.C., Pearson M.L., Silver L.C., Jarvis W.R. (1999). Guideline for prevention of surgical site infection, 1999. Hospital Infection Control Practices Advisory Committee. Infect. Control Hosp. Epidemiol..

[76] National Health and Family Planning Commission of the People’s Republic of China, State Administration of Traditional Chinese Medicine of the People’s Republic of China, Medical Department of the People’s Liberation Army General Logistics Department (2015). Guiding Principles for Clinical Use of Antibiotic (Version 2015) (In Chinese). https://www.nhc.gov.cn/yzygj/s3593/201508/c18e1014de6c45ed9f6f9d592b43db42.shtml.

[77] Beloeil H., Sulpice L. (2016). Peri-operative pain and its consequences. J. Visc. Surg..

[78] Benoist S., Brouquet A. (2015). Nutritional assessment and screening for malnutrition. J. Visc. Surg..

[79] Lew C.C.H., Yandell R., Fraser R.J.L., Chua A.P., Chong M.F.F., Miller M. (2017). Association between Malnutrition and Clinical Outcomes in the Intensive Care Unit: A Systematic Review [Formula: See text]. JPEN J. Parenter. Enter. Nutr..

[80] Skeie E., Tangvik R.J., Nymo L.S., Harthug S., Lassen K., Viste A. (2020). Weight loss and BMI criteria in GLIM’s definition of malnutrition is associated with postoperative complications following abdominal resections—Results from a National Quality Registry. Clin. Nutr..

[81] Kakavas S., Karayiannis D., Bouloubasi Z., Poulia K.A., Kompogiorgas S., Konstantinou D., Vougas V. (2020). Global Leadership Initiative on Malnutrition Criteria Predict Pulmonary Complications and 90-Day Mortality after Major Abdominal Surgery in Cancer Patients. Nutrients.

[82] Beser O.F., Cokugras F.C., Erkan T., Kutlu T., Yagci R.V. (2018). Evaluation of malnutrition development risk in hospitalized children. Nutrition.

[83] Levetan C.S., Passaro M., Jablonski K., Kass M., Ratner R.E. (1998). Unrecognized diabetes among hospitalized patients. Diabetes Care.

[84] Frisch A., Chandra P., Smiley D., Peng L., Rizzo M., Gatcliffe C., Hudson M., Mendoza J., Johnson R., Lin E. (2010). Prevalence and clinical outcome of hyperglycemia in the perioperative period in noncardiac surgery. Diabetes Care.

[85] Smiley D.D., Umpierrez G.E. (2006). Perioperative glucose control in the diabetic or nondiabetic patient. South. Med. J..

[86] Noordzij P.G., Boersma E., Schreiner F., Kertai M.D., Feringa H.H., Dunkelgrun M., Bax J.J., Klein J., Poldermans D. (2007). Increased preoperative glucose levels are associated with perioperative mortality in patients undergoing noncardiac, nonvascular surgery. Eur. J. Endocrinol..

[87] Kwon S., Thompson R., Dellinger P., Yanez D., Farrohki E., Flum D. (2013). Importance of perioperative glycemic control in general surgery: A report from the Surgical Care and Outcomes Assessment Program. Ann. Surg..

[88] Raju T.A., Torjman M.C., Goldberg M.E. (2009). Perioperative blood glucose monitoring in the general surgical population. J. Diabetes Sci. Technol..

[89] Gandhi G.Y., Nuttall G.A., Abel M.D., Mullany C.J., Schaff H.V., Williams B.A., Schrader L.M., Rizza R.A., McMahon M.M. (2005). Intraoperative hyperglycemia and perioperative outcomes in cardiac surgery patients. Mayo Clin. Proc..

[90] Himes C.P., Ganesh R., Wight E.C., Simha V., Liebow M. (2020). Perioperative Evaluation and Management of Endocrine Disorders. Mayo Clin. Proc..

[91] Sizemore D.C., Singh A., Dua A., Singh K., Grose B.W. (2022). Postoperative Nausea. StatPearls, © 2023.

[92] De Lange E., Verhaak P.F., van der Meer K. (2013). Prevalence, presentation and prognosis of delirium in older people in the population, at home and in long term care: A review. Int. J. Geriatr. Psychiatry.

[93] Siddiqi N., House A.O., Holmes J.D. (2006). Occurrence and outcome of delirium in medical in-patients: A systematic literature review. Age Ageing.

[94] Inouye S.K., Westendorp R.G., Saczynski J.S. (2014). Delirium in elderly people. Lancet.

[95] Lovely J.K., Hyland S.J., Smith A.N., Nelson G., Ljungqvist O., Parrish R.H. (2019). Clinical pharmacist perspectives for optimizing pharmacotherapy within Enhanced Recovery after Surgery (ERAS((R))) programs. Int. J. Surg..

[96] Ljungqvist O., Thanh N.X., Nelson G. (2017). ERAS-Value based surgery. J. Surg. Oncol..

[97] Lovely J.K., Larson D.W., Quast J.M. (2014). A clinical practice agreement between pharmacists and surgeons streamlines medication management. Jt. Comm. J. Qual. Patient Saf..

[98] Hammond R.W., Schwartz A.H., Campbell M.J., Remington T.L., Chuck S., Blair M.M., Vassey A.M., Rospond R.M., Herner S.J., Webb C.E. (2003). Collaborative drug therapy management by pharmacists—2003. Pharmacotherapy.

[99] Dobesh P.P., Trujillo T.C., Finks S.W. (2013). Role of the pharmacist in achieving performance measures to improve the prevention and treatment of venous thromboembolism. Pharmacotherapy.

[100] Louzon P., Jennings H., Ali M., Kraisinger M. (2017). Impact of pharmacist management of pain, agitation, and delirium in the intensive care unit through participation in multidisciplinary bundle rounds. Am. J. Health Syst. Pharm. AJHP Off. J. Am. Soc. Health Syst. Pharm..

[101] Neville H.L., Chevalier B., Daley C., Nodwell L., Harding C., Hiltz A., MacDonald T., Skedgel C., MacKinnon N.J., Slayter K. (2014). Clinical benefits and economic impact of post-surgical care provided by pharmacists in a Canadian hospital. Int. J. Pharm. Pract..

[102] Charpiat B., Goutelle S., Schoeffler M., Aubrun F., Viale J.P., Ducerf C., Leboucher G., Allenet B. (2012). Prescriptions analysis by clinical pharmacists in the post-operative period: A 4-year prospective study. Acta Anaesthesiol. Scand..

[103] Nguyen A.D., Lam A., Banakh I., Lam S., Crofts T. (2020). Improved Medication Management with Introduction of a Perioperative and Prescribing Pharmacist Service. J. Pharm. Pract..

[104] Beckman E.J., Hovey S., Bondi D.S., Patel G., Parrish R.H. (2022). Pediatric Perioperative Clinical Pharmacy Practice: Clinical Considerations and Management: An Opinion of the Pediatrics and Perioperative Care Practice and Research Networks of the American College of Clinical Pharmacy. J. Pediatr. Pharmacol. Ther. JPPT Off. J. PPAG.

[105] Rove K.O., Edney J.C., Brockel M.A. (2018). Enhanced recovery after surgery in children: Promising, evidence-based multidisciplinary care. Pediatr. Anesth..

[106] Fernandes B.D., Ribeiro L.C., Pereira dos Santos J.C., Ayres L.R., Chemello C. (2021). Medication Reconciliation at hospital admission and discharge: Evaluation of fidelity and process outcomes in a real-world setting. Int. J. Clin. Pract..

[107] Zheng X., Xiao L., Li Y., Qiu F., Huang W., Li X. (2022). Improving safety and efficacy with pharmacist medication reconciliation in orthopedic joint surgery within an enhanced recovery after surgery program. BMC Health Serv. Res..

[108] Guisado-Gil A.B., Ramirez-Duque N., Baron-Franco B., Sanchez-Hidalgo M., De la Portilla F., Santos-Rubio M.D. (2021). Impact of a multidisciplinary medication reconciliation program on clinical outcomes: A pre-post intervention study in surgical patients. Res. Soc. Adm. Pharm. RSAP.

[109] Akamine A., Nagasaki Y., Tomizawa A., Arai M., Atsuda K. (2022). Risk Factors for Non-Adherence to Medications That Affect Surgery: A Retrospective Study in Japan. Patient Prefer. Adherence.

[110] Wang R., Dong X., Zhang X., Gan S., Kong L., Lu X., Rao Y. (2020). Pharmacist-driven multidisciplinary initiative continuously improves postoperative nausea and vomiting in female patients undergoing abdominal surgery. J. Clin. Pharm. Ther..

[111] Khan Y.H., Alzarea A.I., Alotaibi N.H., Alatawi A.D., Khokhar A., Alanazi A.S., Butt M.H., Alshehri A.A., Alshehri S., Alatawi Y. (2022). Evaluation of Impact of a Pharmacist-Led Educational Campaign on Disease Knowledge, Practices and Medication Adherence for Type-2 Diabetic Patients: A Prospective Pre- and Post-Analysis. Int. J. Environ. Res. Public Health.

[112] Zhuo Y., Pan Y., Lin K., Yin G., Wu Y., Xu J., Cai D., Xu L. (2022). Effectiveness of clinical pharmacist-led smartphone application on medication adherence, insulin injection technique and glycemic control for women with gestational diabetes receiving multiple daily insulin injection: A randomized clinical trial. Prim. Care Diabetes.

[113] Poonprapai P., Lerkiatbundit S., Saengcharoen W. (2022). Family support-based intervention using a mobile application provided by pharmacists for older adults with diabetes to improve glycaemic control: A randomised controlled trial. Int. J. Clin. Pharm..

[114] Shahrami B., Sefidani Forough A., Najmeddin F., Hadidi E., Toomaj S., Javadi M.R., Gholami K., Sadeghi K. (2022). Identification of drug-related problems followed by clinical pharmacist interventions in an outpatient pharmacotherapy clinic. J. Clin. Pharm. Ther..

[115] Peasah S.K., Hammond T., Campbell V., Liu Y., Morgan M., Kearney S., Good C.B. (2022). Assessing the impact of adding pharmacist management services to an existing discharge planning program on 30-day readmissions. J. Am. Pharm. Assoc. JAPhA.

[116] Van Lieshout J., Lacroix J., van Halteren A., Teichert M. (2022). Effectiveness of a Pharmacist-Led Web-Based Medication Adherence Tool with Patient-Centered Communication: Results of a Clustered Randomized Controlled Trial. J. Med. Internet Res..

